# Pathogenicity of *Aspergillus* Airborne Fungal Species Collected from Indoor and Outdoor Public Areas in Tianjin, China

**DOI:** 10.3390/pathogens12091154

**Published:** 2023-09-11

**Authors:** Md M. H. Nafis, Ziwei M. Quach, Amran A. Q. A. Al-Shaarani, Mohammed H. M. Muafa, Lorenzo Pecoraro

**Affiliations:** School of Pharmaceutical Science and Technology, Tianjin University, 92 Weijin Road, Tianjin 300072, China

**Keywords:** airborne fungi, *Aspergillus* species, *Drosophila melanogaster* model, fungal pathogenicity, human health risk assessment, ITS sequencing, microbial air pollution, morphology

## Abstract

Airborne fungi play an important role in air pollution and may have various negative effects on human health. In particular, *Aspergillus* fungi are pathogenic to humans and several domestic animals. In this work, *Aspergillus* strains isolated from airborne fungal communities sampled from different indoor and outdoor environments in Tianjin University were tested for pathogenicity on *Drosophila melanogaster*. Airborne fungi were sampled using an HAS-100B air sampler, over a one-year sampling period. Isolated fungal strains were identified based on morphological and molecular analysis. The *Aspergillus*-centered study was conducted as part of a larger work focusing on the total airborne fungal community in the analyzed environments, which yielded 173 fungal species. In this context, the genus *Aspergillus* showed the second-highest species richness, with 14 isolated species. Pathogenicity tests performed on male adults of *Drosophila melanogaster* through a bodily contact bioassay showed that all analyzed airborne *Aspergillus* species were pathogenic to fruit flies, with high insect mortality rates and shortened lifespan. All the studied fungi induced 100% mortality of fruit flies within 30 culture days, with one exception constituted by *A. creber* (39 days), while the shortest lifespan (17 days) was observed in fruit flies treated with *A. tubingensis*. Our results allow us to hypothesize that the studied airborne fungal species may have a pathogenic effect on humans, given the affinity between fruit flies and the human immune system, and may help to explain the health risk linked with *Aspergillus* fungi exposure in densely populated environments.

## 1. Introduction

Fungi are eukaryotic organisms defined by their heterotrophic means of acquiring nutrition from the environment [1]. They have been found to thrive in a large variety of environments, with many species being colonizers and parasites of plants and animals [2]. Since spores of various fungal species are dispersed in the atmosphere, airborne fungi play an important role in air pollution [3,4,5]. The diameter of airborne fungal spores normally ranges from 2 to 50 μm [6], which allows them to relatively easily penetrate the lower airways of the human respiratory tract, thus resulting in unavoidable inhalation, with consequent allergic sensitization caused by more than 80 genera of fungi [7]. The allergenic and pathogenic potential of a wide range of fungal taxa has been described. For example, the airborne fungal spores of *Alternaria*, *Aspergillus*, and *Cladosporium* have been found to be responsible for a number of respiratory diseases [8]. In particular, *Aspergillus* fungi are pathogenic to several animal species, including humans and domestic animals, and plants. For instance, *A. flavus* is a latent fungal pathogen that causes invasive aspergillosis, usually fatal infections in humans, by colonizing the lungs or respiratory tract. Patients infected with *A. flavus* often show reduced or compromised immune systems [9]. *Aspergillus fumigatus* can cause a range of pulmonary diseases, being one of the most common airborne fungal pathogens in humans, responsible for 90% of all invasive aspergillosis cases [10]. The clinical features of *Aspergillus* infections depend on the interplay between the fungi and the host, which is largely influenced by the host’s immune status [11]. Because people are frequently exposed to *Aspergillus* fungal spores in the majority of indoor and outdoor environments, it is of crucial importance to consider the associated health risk [12].

Recently, many studies have used fruit flies (*Drosophila melanogaster*) as model organisms to test the pathogenicity of fungal species [13]. Fruit flies are an attractive model for studying host–pathogen interactions, since their immune system shares traits with the mammalian innate immune system [14]. Indeed, fruit fly models have received widespread attention due to the high degree of gene conservation between these insects and humans, with 75% of human disease-related genes having corresponding genes in fruit flies [15,16,17]. The *Drosophila melanogaster* model has been recently used to analyze various human medical issues. For instance, a recent study performed by Wang et al. [18] showed that the Malpighian tubules in the renal system of fruit flies are similar to the mammalian renal tubules in both function and structure, pointing out that the kidney stone models of the fruit flies can supply quicker, cost-limited, and high-throughput screening platforms for the development of novel drugs to treat kidney stones [15]. Fruit flies have become an excellent model for cost-effectively and expediently studying nutrient-sensing pathways and metabolic homeostasis [18,19]. In a study conducted by Rani et al. [20], Malpighian tubules of *Drosophila* were used as a model to investigate the effects of chronic exposure to a high-sugar diet on renal tubules [21]. The embryotic, larval, and adult hearts of *Drosophila* are considered good models for studying human cardiovascular diseases, such as arrhythmia [22] and cardiomyopathy [11]. In addition, the life cycle of fruit flies, including the four developmental stages of embryo, larva, pupa, and adult, is very rapid compared with mammals, thus making these insects of great practical use for experiments. At room temperature, one pair of mating flies can produce hundreds of offspring within 12 days [15]. Lower breeding costs, a shorter experiment period, better-understood genome, and more abundant genetic modification techniques make the fruit fly a promising animal model for fundamental genetic studies, large-scale in vivo drug screening and high-throughput screening of putative fungal species virulence factors [20]. In a study conducted by [13], *Drosophila melanogaster* was used as a model host to assess the pathogenicity of *Aspergillus flavus* strains derived from clinical sources, showing that while all analyzed fungal strains were virulent, some of them were characterized by a significantly higher level of virulence, leading to a wide mortality rate in the tested insects [23]. The fungal species *A. terreus*, which is often reported as a cause of invasive aspergillosis, was observed to be able to infect the Toll-deficient (Tl^−/−^) *D. melanogaster* by [24].

At present, it is unclear whether different *Aspergillus* strains belonging to various species, such as *A. oryzae* and *A. versicolor*, vary in their virulence on humans and other animals. In this work, *Aspergillus* strains isolated from airborne fungal communities sampled from different indoor and outdoor environments in Tianjin University, Tianjin, China, were tested for pathogenicity on *Drosophila melanogaster* in order to understand the human health risk associated with the exposure to *Aspergillus* fungi present in the air of a densely populated public environment.

## 2. Materials and Methods

### 2.1. Sampling and Fungal Identification

Sampling of airborne fungi was carried out in Tianjin University campus, Tianjin city, China. This study was conducted as part of a larger work, which analyzed the whole airborne fungal community in the selected university environments [25]. Six different sites, including one outdoor and five indoor environments, were selected to collect the air samples (see Yuan et al., 2022 [25] for details). The sampling was conducted every month, from June 2020 to May 2021. Air samples were collected using an air sampler (HAS-100B) (Hengao T&D, Beijing, China), which was operated at a flow rate of 100 L/min and rotating dish speed of 0–4 rpm for 10 min at each study site. A Petri dish (9.0 cm diameter) containing Malt Extract Agar (MEA), added with ampicillin to prevent bacterial growth, was used for each air sampling. In order to understand the human traffic influence on the airborne fungal community, two different sampling times were chosen, including (i) the off-peak period, when there were no or there were very few human activities, and (ii) the peak period, when there were maximum levels of human presence and activities. After sampling, the plates were put into an incubator at 25 °C in the darkness for 5–7 days and examined every day for fungal growth. For isolation, observed fungal colonies in each plate were picked up and inoculated in new plates (6.0 cm diameter). 

All the isolated *Aspergillus* strains identified using molecular and morphological analyses were selected for subsequent pathogenicity tests. Fungal morphology was characterized based on macroscopic and microscopic observations. Microscopy was carried out using a Nikon ECLIPSE Ci-L microscope (Tokyo, Japan) to examine fungal morphological characteristics, including hyphae, conidiophores, conidia, etc. [26,27,28]. For molecular identification, DNA extraction of *Aspergillus* strains was conducted based on the cetyltrimethylammonium bromide method [29,30]. Fungal ITS regions were amplified via polymerase chain reaction (PCR), using the primers ITS1 and ITS4 [31]. Amplifications were performed following the conditions described in Yuan et al. [25]. The resulting PCR products were electrophoresed in 1% agarose gel and purified with the QIAEX II Gel Extraction Kit (QIAGEN) following the manufacturer’s instructions. DNA sequencing was performed at Tsingke Biological Technology Company (Beijing, China). Sequences were edited, assembled using the program Sequencher 4.1 for MacOS X, and analyzed with BLAST searches against the National Center for Biotechnology Information (NCBI) sequence database (http://www.ncbi.nlm.nih.gov/Blast.cgi, accessed on 25 March 2023). Fungal DNA sequences amplified from the isolated *Aspergillus* stains were submitted to GenBank under accessions OM237038, OM236862, OM237112, OM237034, OM236745, OM236800, OM236675, OM237191, OM236713, OM236847, OM237054, OM237070, OM236676, and OM236712.

Phylogenetic analysis was conducted with Mega v. 7.0 [32], DNA sequences were aligned with Clustal X v. 2.1 [33], and both a neighbour-joining tree and a maximum likelihood tree against selected database sequences were constructed using Kimura 2-parameter distances, with bootstrapping of 1000 replicates [34]. *Metarhizium anisopliae* was used as outgroup to root the *Aspergillus* fungi trees.

All analyzed fungal strains were deposited in the LP Culture Collection (personal culture collection held in the laboratory of Prof. Lorenzo Pecoraro), at the School of Pharmaceutical Science and Technology, Tianjin University, Tianjin, China.

### 2.2. Drosophila Maintenance

Pathogenicity tests were performed on male adults of *Drosophila melanogaster* to minimize sex-dependent effects on susceptibility to infection. Wild-type (WT) Dijon 2000 (*Di2*) flies were used because they are less laborious to manipulate compared to other types [35]. Fly reproduction was carried out in an insectary under controlled temperature conditions (25 °C). *Drosophila melanogaster* individuals were kept in transparent bottles (150 mL) containing 30 mL of nutrient medium constituted by yeast 24.5 g, corn 50 g, agar 10 g, sucrose 7.25 g, brown sugar 30 g, 4 mL propionic acid, and 10% antibiotic (5.1 g Methyl 4-hydroxybenzoate dissolved in 50 mL absolute alcohol × 3) in 1 L of distilled water. The bottles of fruit flies with the nutrient medium were maintained at 25 °C temperature under a 12 h dark/12 h light cycle. 

### 2.3. Pathogenicity Test

**Figure 1:**
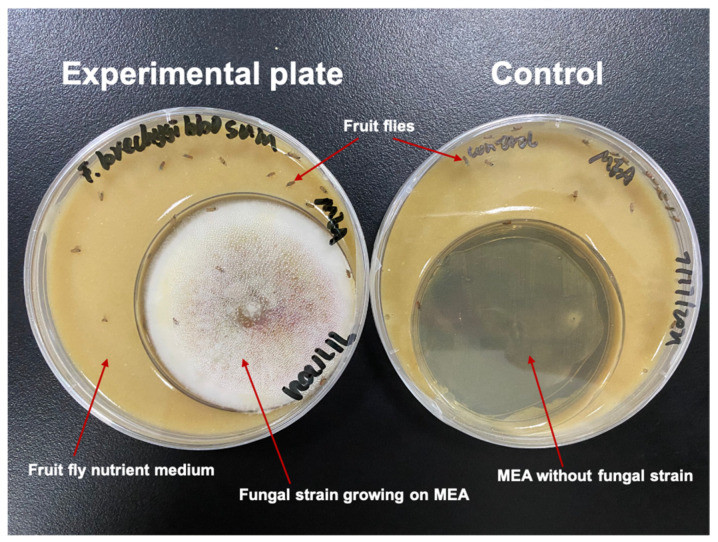
Example of pathogenicity test. In the experimental plate (**left**), the fruit flies were exposed to the fungus and could feed on the nutrient medium. In the control plate (**right**), the smaller capsule only contained MEA, without fungal inoculum.

A bodily contact bioassay tested the pathogenicity of the airborne *Aspergillus* isolates toward fruit flies. *Aspergillus* isolates were grown on Malt Extract Agar (MEA) in small Petri dishes (6.0 cm diameter), which were placed inside larger Petri dishes (9.0 cm diameter) filled with the fruit fly nutrient medium (Figure 1). In such experimental conditions, the fruit flies were in constant contact, during the whole period of observation, with a source of fungal particles represented by the same surface of actively growing and sporulating mycelium for each fungal strain, which was cultivated under exactly the same environmental parameters, for the same number of days, in order to make the experiments homogeneous and reliable. To identify the male *D. melanogaster* from the individuals contained in the transparent bottles, we used a stereoscopic microscope equipped with a controllable CO2-flow fly pad. A sterilized brush was used to separate the flies under the microscope, and 20 anaesthetized male flies were placed into each Petri dish prepared with the nutrient medium and the selected *Aspergillus* strain. We prepared 5 plate replicates to test each fungal strain and 1 control plate (without fungi). The experimental plates were kept at room temperature 25 °C and monitored every 24 h to detect fly mortality. The daily number of dead flies was recorded until all individuals were dead. Fungal growth was observed daily on dead flies, until fungal sporulation was visible, using a Nikon ECLIPSE Ci-L microscope (Tokyo, Japan) to verify that the tested fungi were responsible for the observed *Drosophila* individuals’ mortality.

## 3. Results

### 3.1. Diversity of Aspergillus Strains Isolated from the Analyzed Air Environments

This study focused on *Aspergillus* fungi as members of the total airborne fungal community constituted by 173 species in 74 genera and 56 families, out of 641 fungal strains isolated during a larger work performed by our research group in the six investigated locations at Tianjin University campus [25]. In the context of the whole analyzed airborne fungal community, the genus *Aspergillus* showed the second-highest species richness, with 14 species isolated from different locations, months, and levels of human activity (Table 1). Within the genus *Aspergillus*, *A. flavus* was the most abundant species, with seven strains isolated from the studied air environments. 

**Figure 2:**
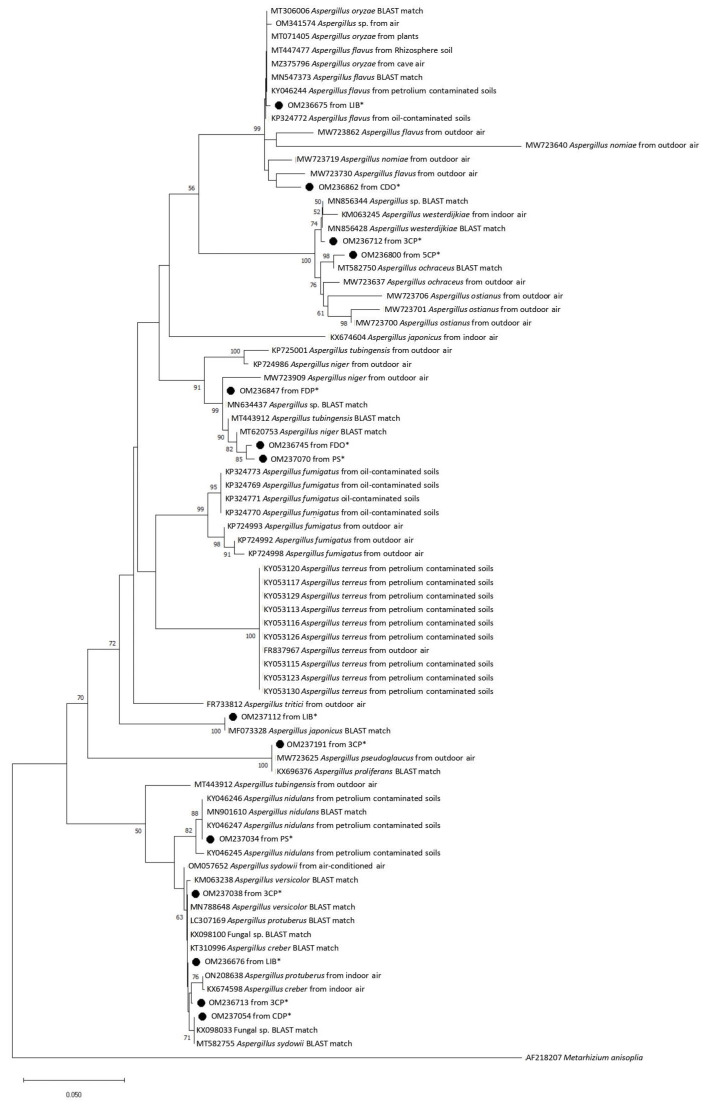
Neighbour-joining phylogenetic tree showing the relationship between the *Aspergillus* sequences obtained from Tianjin University campus airborne fungal communities (*) and selected database relatives. Kimura 2-parameter distances were used. Bootstrap values were based on percentages of 1000 replicates. *Metarhizium anisopliae* (AF218207) was used as the outgroup. 3CP = Canteen 3 Peak, 3CO = Canteen 3 Off-peak, 5CP = Canteen 5 Peak, 5CO = Canteen 5 Off-peak, CDP = Chinese students Dorm Peak, CDO = Chinese students Dorm Off-peak, FDP = Foreign students Dorm Peak, FDO = Foreign students Dorm Off-peak, LIB = Library, PS = Peiyang Square.

**Figure 3:**
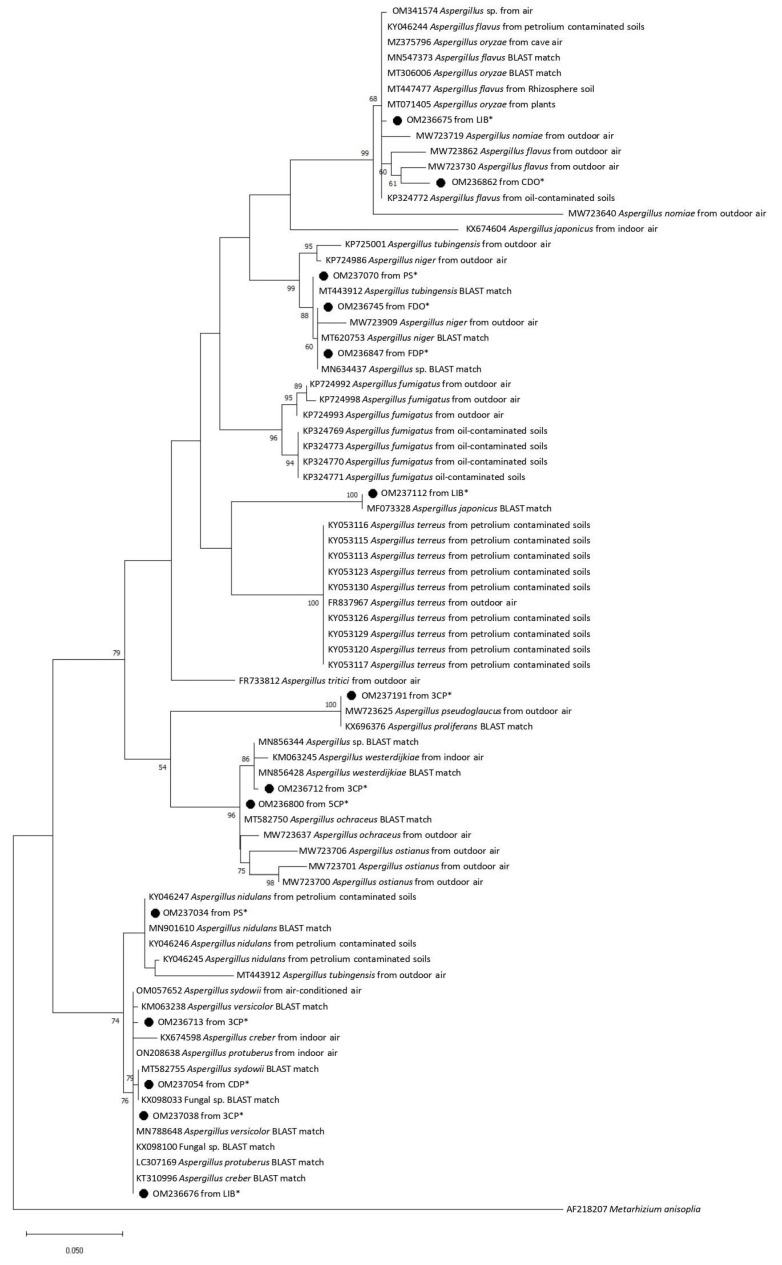
Maximum likelihood phylogenetic tree showing the relationship between the *Aspergillus* sequences obtained from Tianjin University campus airborne fungal communities (*) and selected database relatives. Kimura 2-parameter distances were used. Bootstrap values were based on percentages of 1000 replicates. *Metarhizium anisopliae* (AF218207) was used as the outgroup. 3CP = Canteen 3 Peak, 3CO = Canteen 3 Off-peak, 5CP = Canteen 5 Peak, 5CO = Canteen 5 Off-peak, CDP = Chinese students Dorm Peak, CDO = Chinese students Dorm Off-peak, FDP = Foreign students Dorm Peak, FDO = Foreign students Dorm Off-peak, LIB = Library, PS = Peiyang Square.

Phylogenetic analysis clarified the relationship of airborne fungi isolated from indoor and outdoor environments in Tianjin University within the genus *Aspergillus*. Sequences retrieved from the university campus-analyzed strains could be aligned with GenBank sequences from various *Aspergillus* species collected in different environments and countries, including indoor and outdoor air in China, Slovakia, Turkey, Czech Republic, and Korea, oil-contaminated soils, and plant tissues. Both in the neighbor-joining tree and in the maximum likelihood tree from the *Aspergillus* dataset (Figure 2 and Figure 3), the sequence OM236675 from a strain isolated in the library of Tianjin University fell in a cluster, including *A. flavus* and *A. oryzae*. The sequence OM236862 obtained from a strain collected in a Chinese student’s dorm during the off-peak period was phylogenetically close to *A. flavus* previously found in outdoor air in China. The fungal sequence OM236712 obtained from canteen 3 at the peak period was segregated in a cluster with GenBank sequences from *A. westerdijkiae*. The three sequences OM236800 from canteen 5 at the peak period, OM236847 from a foreign student’s dorm at the peak period, and OM237112 from the library formed clades with their best BLAST matches, *A. ochraceus* MT582750, *Aspergillus* sp. MN634437, and *A. japonicus* MF073328, respectively. The fungal sequences OM236745, amplified from a foreign student’s dorm off-peak period, and OM237070 from Peiyang Square, co-segregated into a cluster included their best BLAST matches *A. niger* MT620753 and *A. tubingensis* MT443912 (Figure 2 and Figure 3, Table 1). The sequence OM237191 from the airborne fungal strain isolated from canteen 3 during the peak period clustered with its best BLAST match *A. proliferans* (KX696376) and *A. pseudoglaucus* previously found in outdoor air in China. The sequence OM237038 retrieved from canteen 3 during the peak period fell in a cluster with its best BLAST matches, Fungal sp. KX098100 and *A. creber* KT310996, as well as with other GenBank *Aspergillus* sequences. The sequence OM237034 from a strain isolated in Peiyang Square clustered with *A. nidulans*, while the sequence OM236676 collected from the library showed phylogenetic relationships with several GenBank *Aspergillus* sequences, including its best BLAST match *A. versicolor*. The sequence isolated from the canteen 3 peak period (OM236713) clustered with *A. protuberus* and *A. creber* sequences previously collected from indoor air in China and Turkey, respectively. The sequence OM237054 retrieved from a Chinese student’s dorm at the peak period was closely related to *A. sydowii*.

### 3.2. Pathogenicity of Aspergillus Fungi on Fruit Flies

**Figure 4:**
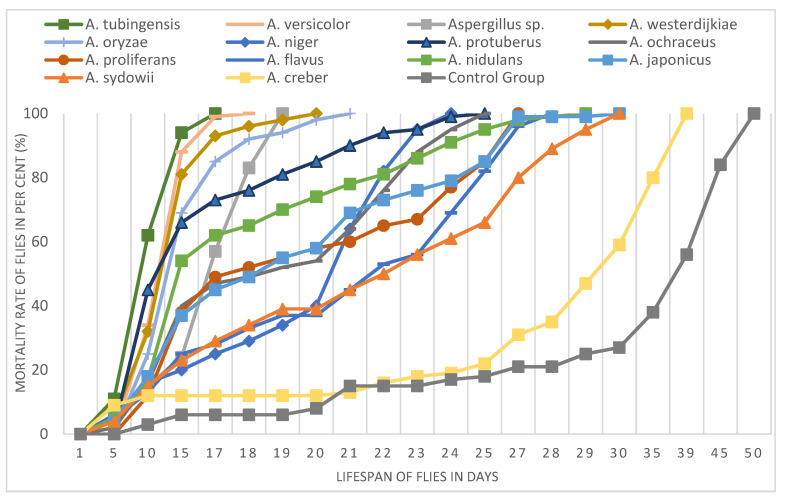
Lifespan (days) and mortality rate (%) of fruit flies (*Drosophila melanogaster*) treated with *Aspergillus* species.

The analyzed *Aspergillus* species displayed a different effect on the *Drosophila* fly lifespan (Figure 4). All the studied fungi induced 100% mortality of fruit flies within 30 culture days, with one exception constituted by *A. creber*. *Drosophila* individuals treated with the latter *Aspergillus* species showed the longest lifespan (39 days), while the shortest lifespan (17 days) was observed in fruit flies treated with *A. tubingensis*. The fruit fly’s lifespan in the control group was 50 days, which was considerably longer than in all 14 tested *Aspergillus* species, thus demonstrating the efficacy of the bodily contact bioassay technique of inoculation. 

**Figure 5:**
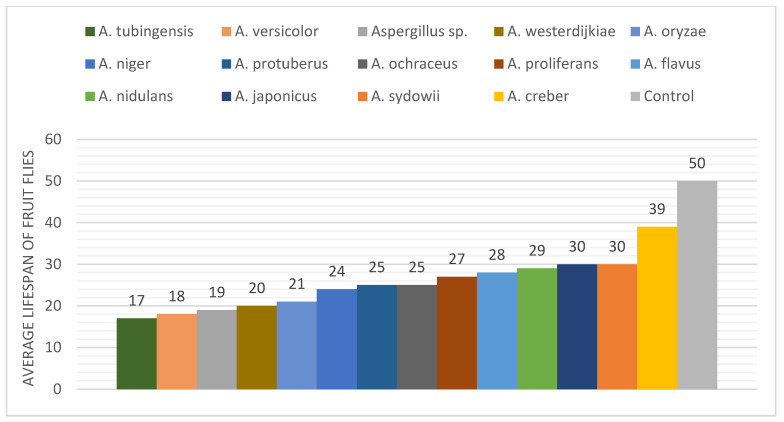
Lifespan of fruit flies (*Drosophila melanogaster*) treated with *Aspergillus* fungi.

Some fungal species showed an identical effect on the fruit fly lifespan. For instance, *A. protuberus* and *A. ochraceus* shortened the insect lifespan to 25 days, while *A. japonicus* and *A. sydowii* produced 100% mortality in the model organism after 30 days (Figure 5). *Aspergillus versicolor*, *Aspergillus* sp., *A. westerdijkiae*, and *A. oryzae* showed a similar shortening effect on *Drosophila* lifespan (18, 19, 20, and 21 days, respectively). *Aspergillus proliferans*, *A. flavus*, and *A. nidulans* produced a similar effect by reducing the fruit fly lifespan to 27, 28, and 29 days, respectively.

**Figure 6:**
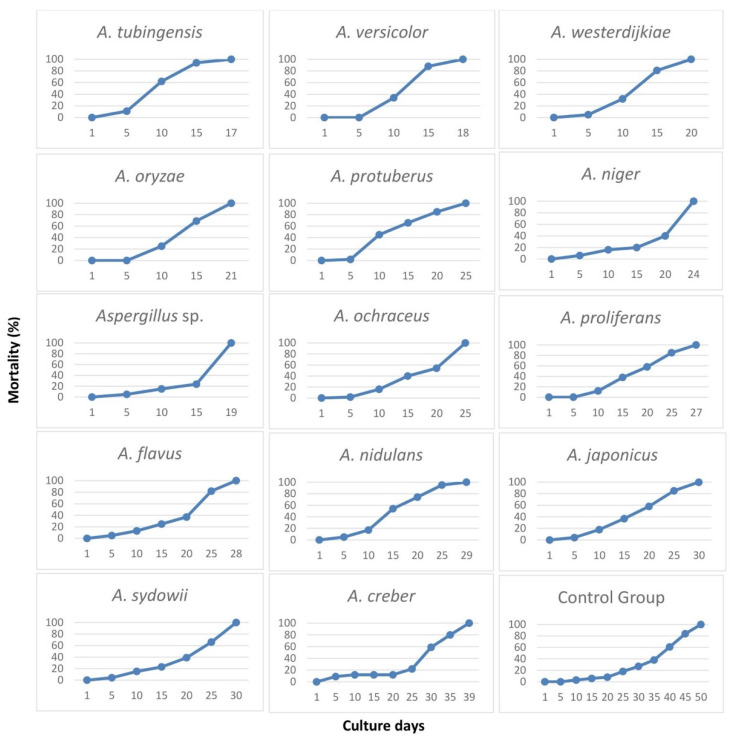
Mortality (%) of fruit flies (*Drosophila melanogaster*) treated with *Aspergillus* fungi, in five-days intervals.

The mortality ratio of fruit flies was observed and recorded every day. We summarized the results with 5-day intervals in Table 2. The analyzed *Aspergillus* species induced different death ratios on fruit flies. In most cases, the mortality rate was within 5% after five culture days, except for *A. tubingensis*, *A. creber*, and *A. niger* that produced 11%, 9%, and 6% mortality rates, respectively (Table 2). The earliest death of fruit flies was recorded with *A. creber* after 3 days. No mortality was recorded during the first five culture days for the fruit flies treated with *A. versicolor*, *A. oryzae*, and *A. proliferans*. After culture day 10, for most of the *Aspergillus* species, the *Drosophila* mortality rate was less than 20%, except for *A. tubingensis*, *A. protuberus*, *A. versicolor*, *A. westerdijkiae*, and *A. oryzae*, which showed significantly higher mortality rates (62%, 45%, 34%, 32%, and 25%, respectively). The mortality rate of flies for *A. tubingensis*, *A. versicolor*, and *A. westerdijkiae* was higher than 80% after 15 days. *Aspergillus* sp. showed a remarkable increase in the fly death rate, from 24% to 100%, in just 4 days, after culture day 15 (Figure 6). The four fungal species *A. tubingensis*, *A. versicolor*, *Aspergillus* sp., and *A. westerdijkiae* induced 100% mortality of flies in 20 culture days, while the control group only showed 8% mortality in the same period. For *A. protuberus* and *A. ochraceus* strains, 100% mortality of flies was reached in 25 culture days, whereas the mortality after the same number of culture days was slightly lower with *A. tubingensis* (95%) and considerably lower with *A. proliferans*, *A. japonicus*, and *A. flavus* (82–85%). After 30 culture days, no *Drosophila* individuals survived with the tested *Aspergillus* species, with the sole exception represented by *A. creber*, which only induced 59% mortality in the treated insects in a month. After 35 culture days, the fruit fly death rate was 80% in the presence of *A. creber* and 38% in the control group. 

**Figure 7:**
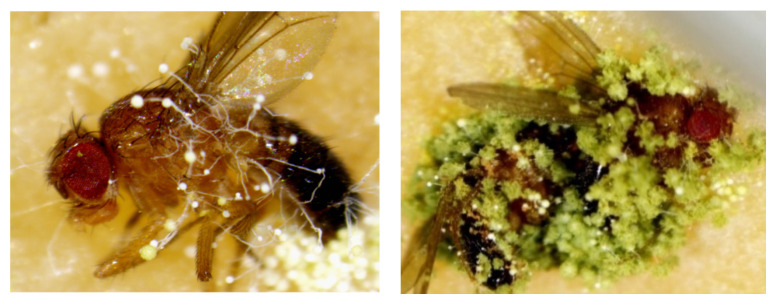
*Aspergillus flavus* sporulating on dead wild-type *Drosophila melanogaster* individuals.

In most cases, dead flies inoculated with *Aspergillus* species showed fungal growth on their bodies, while no external presence of fungi was observed on the flies exposed to *Aspergillus* sp. and *A. sydowii* after death. More specifically, fruit flies treated with *A. tubingensis*, *A. versicolor*, *A. westerdijkiae*, *A. oryzae*, *A. niger*, and *A. flavus* displayed clearly visible fungal colonies 3–4 days after death (Figure 7), while *A. protuberus*, *A. ochraceus*, *A. proliferans*, *A. nidulans*, *A. japonicus*, and *A. creber* hyphal growth was recorded on dead flies after 7–8 days.

## 4. Discussion

This study shed light on the pathogenicity of airborne *Aspergillus* fungal strains isolated from indoor and outdoor environments in Tianjin University campus, a densely populated major university area located in the third-largest municipality of North China [36]. The use of *Drosophila melanogaster* as a model organism in the performed fungal pathogenicity experiments allowed us to hypothesize the risk for human health associated with the presence of the analyzed fungi in the air, given the remarkable affinities in immune systems and disease-related genes between fruit flies and humans [14,17]. All tested *Aspergillus* fungi consistently reduced the lifespan of fruit flies when compared to the control group, with significant variability in pathogenicity observed among the different analyzed fungal species.

The genus *Aspergillus* includes about 339 species, more than 40 of them being known as opportunistic pathogens to plants, animals, and humans [37,38]. In our study, fruit flies treated with *A. tubingensis* showed the shortest lifespan and highest mortality rate. Strong pathogenicity was previously observed by Zahran et al. (2017) [39] using an *A. tubingensis* isolate collected from dead bed bugs, which showed rapid mortality of the tested *Cimex hemipterus* insects from day 6 to day 12, ranging from 13% to 90%, depending on the different doses of the inoculated fungus. In addition, *Aspergillus tubingensis* was described as an opportunistic pathogen in an immunocompromised patient with maxillary osteomyelitis [40]. *Aspergillus versicolor* exhibited a shortening effect on fruit fly lifespan similar to that of *A. tubingensis*. In a previous study, mice exposed to *A. versicolor* aerosol inhalation for 15–20 min showed upper respiratory tract irritation [41]. This fungal species is also known as a plant pathogen, causing fruit rot disease in tomatoes and sour rot disease in grapes [42,43], while it was reported as a post-harvest pathogen on jute seeds [44]. Among the tested *Aspergillus* species, *A. westerdikijae* was found to be the fourth most virulent to fruit flies. A study conducted by Baggio et al. (2015) [45] reported that *A. westerdijkiae* was pathogenic to adult females and oothecae of the American cockroach (*Periplaneta americana*, Blattidae). This fungus is also known to produce ochratoxin A (OTA) as a contaminant in coffee beans in many parts of the world, including Vietnam [46] and Thailand [47]. The mycotoxin OTA, produced by the secondary metabolism of numerous filamentous fungal species, can have various harmful consequences in mammals [48]. Another of our tested *Aspergillus* species, *A. oryzae*, which showed pathogenicity on *D. melanogaster*, was previously recorded for the first time as an entomopathogenic fungus on locusts (*Locusta migratoria*) by Zhang et al. [49]. *Aspergillus niger* and *A. flavus*, two common fungal pathogens on plants and animals [50], confirmed their pathogenicity in our work by drastically shortening the lifespan of *Drosophila melanogaster* fruit flies. Our results are in agreement with previous findings by Ramírez-Camejo et al. [51], who showed pathogenic effects of *A. niger* and *A. flavus* in humans, using the same model organism. All five above-mentioned *Aspergillus* species (*A. tubingensis*, *A. versicolor*, *A. westerdijkiae*, *A. niger*, and *A. flavus*) were previously found during screening for potentially pathogenic isolates from problematic buildings with moisture damage or complaints about indoor air quality in Helsinki, Finland [52]. *Aspergillus protuberus* and *A. ochraceus* showed a similar shortening effect on the lifespan of fruit flies in this study [53] discovered that *A. protuberus* caused a rare case of kerion-type scalp mycosis in humans, whereas a study conducted by Castillo et al. (2000) [54] revealed that *A. ochraceus* significantly decreased fecundity in the *Ceratitis capitata* fruit fly. The analyzed fungal species *A. proliferans*, *A. nidulans,* and *A. japonicus* induced similar high mortality in male flies in this study. *Aspergillus proliferans* and *A. nidulans* are both known to cause diseases in immunocompromised patients, including onychomycosis, caused by *A. proliferans* [55], and chronic granulomatous disease (CGD), caused by *A. nidulans* [56]. Ford and Friedman [57] showed that *A. japonicus* was pathogenic to mice in a study comparing the relative virulence of several species of *Aspergillus*.

Except for *Aspergillus* sp. and *A. sydowii*, all the pathogenic airborne *Aspergillus* species analyzed in our study showed fungal growth on the dead insects, including conidiophores and conidia, which is a common feature observed in previous studies using *Drosophila* flies to test fungal species pathogenicity [58,59]. In spite of the absence of visible fungal structures on the dead bodies of flies treated with *Aspergillus* sp. and *A. sydowii*, the high mortality ratio and limited lifespan of inoculated flies provided the evidence of the pathogenicity of these two fungal species. A study by Bobadilla-Carrillo et al. [60] reported on the presence of *A. sydowii* as a potential pathogenic fungus in marine and freshwater fish commercial feeds. 

*Aspergillus creber* inoculation resulted in the lowest mortality and least pronounced lifespan-shortening effects on fruit flies in our whole study, although this fungus caused the earliest fruit fly death records among all the analyzed airborne *Aspergillus* species, on culture day 3. In a previous study, *A. creber* was found to produce sterigmatocystin in a stored rice sample [61]. Sterigmatocystin, a polyketide mycotoxin that is also known to contaminate various foods, such as spices, grains, bread, beer, and cheeses, may be responsible for serious health problems in humans and other animals [62]. *Aspergillus creber* was described as a contaminant in a commercial freshwater fish feed [60]. Our control group showed a standard fly mortality rate and lifespan (50 days), with no fungal growth after death, as reported in numerous previous studies [63,64,65], thus confirming the validity of our experimental procedure. 

In conclusion, we discovered a wide range of pathogenicity in different *Aspergillus* species isolated from the air of Tianjin University indoor and outdoor environments. All airborne *Aspergillus* species tested in this study were pathogenic to fruit flies, with high mortality rates and shortened lifespan. Our results allow us to hypothesize that the studied airborne fungal species may have a pathogenic effect on humans, given the affinity between fruit flies and the human immune system, and may help to explain the health risk linked with *Aspergillus* fungi exposure in densely populated environments. The presence and concentration of potential pathogenic fungi, such as *Aspergillus* species, in the air of public areas characterized by high human activity should be monitored in order to determine any possible hazardous effect on human health and to prevent diseases associated with microbial pollution.

## Figures and Tables

**Table 1:** *Aspergillus* species detected in indoor and outdoor environments of Tianjin University, from DNA extracted from isolated strains.

No.	Sampling Month	Site Code	GenBank Code	Best BLAST Match(es)	Accession Code	Overlap Length	% Match
01	Jun	LIB	OM236675	*Aspergillus oryzae*	MT306006	1009	99.46%
02	Jun	LIB	OM236676	Fungal sp.	KX098100	961	99.43%
				*Aspergillus versicolor*	MN788648	955	99.25%
03	Jun	3CP	OM236712	*Aspergillus* sp.	MN856344	989	99.09%
				*Aspergillus westerdijkiae*	MN856428	987	98.92%
04	Jun	3CP	OM236713	*Aspergillus protuberus*	LC307169	959	99.43%
05	Jul	FDO	OM236745	*Aspergillus niger*	MT620753	859	99.79%
06	Jul	5CP	OM236800	*Aspergillus ochraceus*	MT582750	905	99.40%
07	Sep	FDP	OM236847	*Aspergillus* sp.	MN634437	1029	99.82%
08	Sep	CDO	OM236862	*Aspergillus flavus*	MN547373	1088	98.38%
09	Oct	PS	OM237034	*Aspergillus nidulans*	MN901610	985	100.00%
10	Oct	3CP	OM237038	Fungal sp.	KX098100	977	99.63%
				*Aspergillus creber*	KT310996	977	99.63%
11	Oct	CDP	OM237054	Fungal sp.	KX098033	976	99.81%
				*Aspergillus sydowii*	MT582755	974	99.81%
12	Nov	PS	OM237070	*Aspergillus tubingensis*	MT443912	928	99.22%
13	Dec	LIB	OM237112	*Aspergillus japonicus*	MF073328	998	99.82%
14	Feb	3CP	OM237191	*Aspergillus proliferans*	KX696376	900	99.20%

BLAST search closest matches of fungal internal transcribed spacer DNA sequences amplified from Tianjin University airborne isolated *Aspergillus* strains. Strain GenBank accession codes, accession codes for the closest GenBank matches, sequence identity, and overlap of each match are reported. 3CP = Canteen 3 Peak, 3CO = Canteen 3 Off-peak, 5CP = Canteen 5 Peak, 5CO = Canteen 5 Off-peak, CDP = Chinese students Dorm Peak, CDO = Chinese students Dorm Off-peak, FDP = Foreign students Dorm Peak, FDO = Foreign students Dorm Off-peak, LIB = Library, PS = Peiyang Square.

**Table 2:** Effect of *Aspergillus* fungal species on fruit fly (*Drosophila melanogaster*) mortality.

Species Name	Day 5 %	Day 10 %	Day 15 %	Day 20 %	Day 25 %	Day 30 %	Day 35 %
*Aspergillus oryzae*	00	25	69	98	-	-	-
*Aspergillus versicolor*	00	34	88	-	-	-	-
*Aspergillus westerdijkiae*	05	32	81	-	-	-	-
*Aspergillus protuberus*	02	45	66	85	100	-	-
*Aspergillus niger*	06	16	20	40	-	-	-
*Aspergillus ochraceus*	02	16	40	54	100	-	-
*Aspergillus* sp.	05	15	24	-	-	-	-
*Aspergillus flavus*	05	13	25	37	82	-	-
*Aspergillus nidulans*	05	17	54	74	95	-	-
*Aspergillus creber*	09	12	12	12	22	59	80
*Aspergillus sydowii*	04	15	23	39	66	100	-
*Aspergillus tubingensis*	11	62	94	-	-	-	-
*Aspergillus japonicus*	04	18	37	58	85	100	-
*Aspergillus proliferans*	00	12	38	58	85	-	-
Control group	00	03	06	08	18	27	38

Mortality rates are reported in percent (%).

## Data Availability

Data presented in this study can be found as part of the manuscript. The fungal DNA sequences amplified during this study are available at GenBank under accessions OM237038, OM236862, OM237112, OM237034, OM236745, OM236800, OM236675, OM237191, OM236713, OM236847, OM237054, OM237070, OM236676, and OM236712.
